# Structure and Inhibition of the SARS Coronavirus Envelope Protein Ion Channel

**DOI:** 10.1371/journal.ppat.1000511

**Published:** 2009-07-10

**Authors:** Konstantin Pervushin, Edward Tan, Krupakar Parthasarathy, Xin Lin, Feng Li Jiang, Dejie Yu, Ardcharaporn Vararattanavech, Tuck Wah Soong, Ding Xiang Liu, Jaume Torres

**Affiliations:** 1 School of Biological Sciences, Nanyang Technological University, Singapore; 2 Biozentrum of University Basel, Basel, Switzerland; 3 Center for Life Sciences, Department of Physiology, National University of Singapore, Singapore; 4 Institute of Molecular Cell Biology, Proteos, Singapore; University of North Carolina, United States of America

## Abstract

The envelope (E) protein from coronaviruses is a small polypeptide that contains at least one α-helical transmembrane domain. Absence, or inactivation, of E protein results in attenuated viruses, due to alterations in either virion morphology or tropism. Apart from its morphogenetic properties, protein E has been reported to have membrane permeabilizing activity. Further, the drug hexamethylene amiloride (HMA), but not amiloride, inhibited in vitro ion channel activity of some synthetic coronavirus E proteins, and also viral replication. We have previously shown for the coronavirus species responsible for severe acute respiratory syndrome (SARS-CoV) that the transmembrane domain of E protein (ETM) forms pentameric α-helical bundles that are likely responsible for the observed channel activity. Herein, using solution NMR in dodecylphosphatidylcholine micelles and energy minimization, we have obtained a model of this channel which features regular α-helices that form a pentameric left-handed parallel bundle. The drug HMA was found to bind inside the lumen of the channel, at both the C-terminal and the N-terminal openings, and, in contrast to amiloride, induced additional chemical shifts in ETM. Full length SARS-CoV E displayed channel activity when transiently expressed in human embryonic kidney 293 (HEK-293) cells in a whole-cell patch clamp set-up. This activity was significantly reduced by hexamethylene amiloride (HMA), but not by amiloride. The channel structure presented herein provides a possible rationale for inhibition, and a platform for future structure-based drug design of this potential pharmacological target.

## Author Summary

Coronaviruses are viral pathogens that cause a variety of lethal diseases in birds and mammals, and common colds in humans. In 2003, however, an animal coronavirus was able to infect humans and produced severe acute respiratory syndrome (SARS), causing a near pandemic. Such events are likely to reoccur in the future, and new antiviral strategies are necessary. A small coronavirus protein called ‘envelope’ is important for pathogenesis, affecting the formation of the viral envelope and the distribution of the virus in the body. In vitro studies have shown that synthetic coronavirus envelope proteins have channel activity that in some cases has been inhibited by the drug hexamethylene amiloride, but not by amiloride. In the present paper, we have characterized the structure responsible for this channel activity. We have also determined the binding site of the drug hexamethylene amiloride in the channel, and shown that amiloride has only a mild effect on the NMR signals from the protein. The validity of these results is supported using mammalian cells expressing full length SARS-CoV E, where channel activity was inhibited by hexamethylene amiloride, but only mildly by amiloride. The structural model described for this channel provides a valuable insight into coronavirus envelope protein ion channel activity, and could serve as a platform for the development of novel anti-viral drugs.

## Introduction

Coronaviruses (family *Coronaviridae*, genus *Coronavirus* [1]) are enveloped viruses that cause common colds in humans and a variety of lethal diseases in birds and mammals [2]–[4]. The virus species in the genus *Coronavirus* have been organized into 3 groups, using genetic and antigenic criteria [5]. Group 1 is subdivided into two groups, 1a and 1b. Group 1a includes the porcine Transmissible gastroenteritis virus (TGEV), whereas group 1b includes Human coronaviruses 229E (HCoV-229E) or NL63 (HCoV-NL63). Group 2 is also subdivided in groups 2a, e.g., Murine hepatitis virus (MHV) and Human coronavirus OC43 (HCoV-OC43) and 2b, e.g., the virus responsible for the severe acute respiratory syndrome (SARS-CoV) [6],[7]. Group 3 includes the avian Infectious bronchitis virus (IBV) and the turkey coronavirus (TCoV).

SARS-CoV produced a near pandemic in 2003 [8], with 8,096 infected cases and 774 deaths worldwide (http://www.who.int/csr/sarsarchive/2003_05_07a/en/). SARS-CoV was enzootic in an unknown animal or bird species, probably a bat [9], before suddenly emerging as a virulent virus in humans. A similar crossing of the animal-human species barrier is thought to have occurred between the bovine coronavirus (BCoV) and human coronavirus OC43 (HCoV-OC43) more than 100 years ago [10]. Such coronavirus interspecies jumps, from animal hosts to humans, are likely to reoccur in the future.

There is therefore an urgent need to know more about the coronavirus life cycle, and about new ways to battle infection. Protective efficacy of candidate vaccines against coronaviruses in humans has been mainly studied in animals so far, and only few vaccines have entered Phase 1 human trials [11]. Other compounds [12]–[17] have shown activity against SARS-CoV and HCoV-229E, but there is no data from animal studies or clinical trials [18]. Studies of antiviral therapy against coronaviruses other than SARS-CoV have been scarce; in vitro data show that several chemicals may have inhibitory activities on HCoV-NL63 and HCoV-229E [19],[20], but there have not been clinical trials on therapy of infections caused by human coronaviruses HCoV-OC43, HCoV-229E, HCoV-NL63 and HCoV-HKU1.

All coronaviruses express the envelope (E) protein, a typically short polypeptide that in SARS-CoV is 76 amino acids long, and which contains at least one α-helical transmembrane domain (ETM). In SARS-CoV E the transmembrane domain spans ∼25 residues [21], approximately from residue 10 to 35. Coronavirus E proteins are incorporated into the virion lipidic envelope, along with the spike protein (S) and the membrane protein (M). While the S protein is involved in fusion with host membranes during entry into cells, and the M protein is important in envelope formation and budding, E protein is not essential for *in vitro* and *in vivo* coronavirus replication. However, its absence results in an attenuated virus, as shown for SARS-CoV [22]. Recently, using a transgenic mouse model expressing the SARS-CoV receptor human angiotensin converting enzyme-2 (hACE-2), SARS coronavirus lacking gene E was shown to be attenuated and, in contrast to the wild type virus, did not grow in the central nervous system [23]. In other coronaviruses, E protein affects viral morphogenesis, i.e., virus-like particle (VLP) formation and release [24]–[29]. Indeed, mutations in the extramembrane domain of E protein impaired viral assembly and maturation in MHV [30]. In TGEV, the absence of E protein resulted in a blockade of virus trafficking in the secretory pathway and prevention of virus maturation [31],[32].

In addition to the aforementioned roles of E protein in morphogenesis and tropism, enhanced membrane permeability has been observed in bacterial and mammalian cells expressing MHV E [33] or SARS-CoV E [34]. It has also been reported that synthetic E proteins of SARS-CoV, HCoV-229E, MHV, and IBV, have in vitro cation-selective ion channel activity in planar lipid bilayers, and this activity has been shown to be localized at the transmembrane domain [35]–[37]. It was also shown that the drug hexamethylene amiloride (HMA), but not amiloride, inhibited in vitro conductance of synthetic MHV E and HCoV-229E E, and decreased viral replication of MHV and HCoV-229E in infected cells [36].

To determine if this channel activity is biologically relevant, this function must be associated to a structural organization compatible with an ion channel, together with electrophysiological studies performed using the complete polypeptide. Lastly, a correlation between inhibition and a molecular description of drug-channel interaction must be obtained. The data currently available, however, (see above) was obtained using synthetic transmembrane peptides or unpurified synthetic E proteins in non-physiological environments [35]–[37], or using qualitative permeability assays [33],[34], and the target of HMA was not unequivocally determined [36].

The fact that SARS-CoV ETM forms only pentamers in dodecylphosphocholine (DPC) and perfluorooctanoic (PFO) micelles [38], strongly suggests that the ion channel activity of coronavirus E proteins is caused by a pentameric ion channel. Therefore, in the present work our aim was (i) to use NMR to determine the structure of the pentameric oligomer formed by a selectively labeled SARS-CoV ETM (residues 8 to 38) when reconstituted in DPC micelles, (ii) to characterize the interaction of HMA or amiloride with this channel, and (iii) to test if this data is still relevant in a more physiological environment, using patch clamped mammalian cells expressing full length SARS-CoV E. The structural model described for this channel provides a valuable insight into coronavirus envelope ion channel activity, ion selectivity and channel inhibition, and could serve as a platform for the development of novel anti-viral drugs.

## Results

### 3D structure of the ETM channel

**Figure 1:**
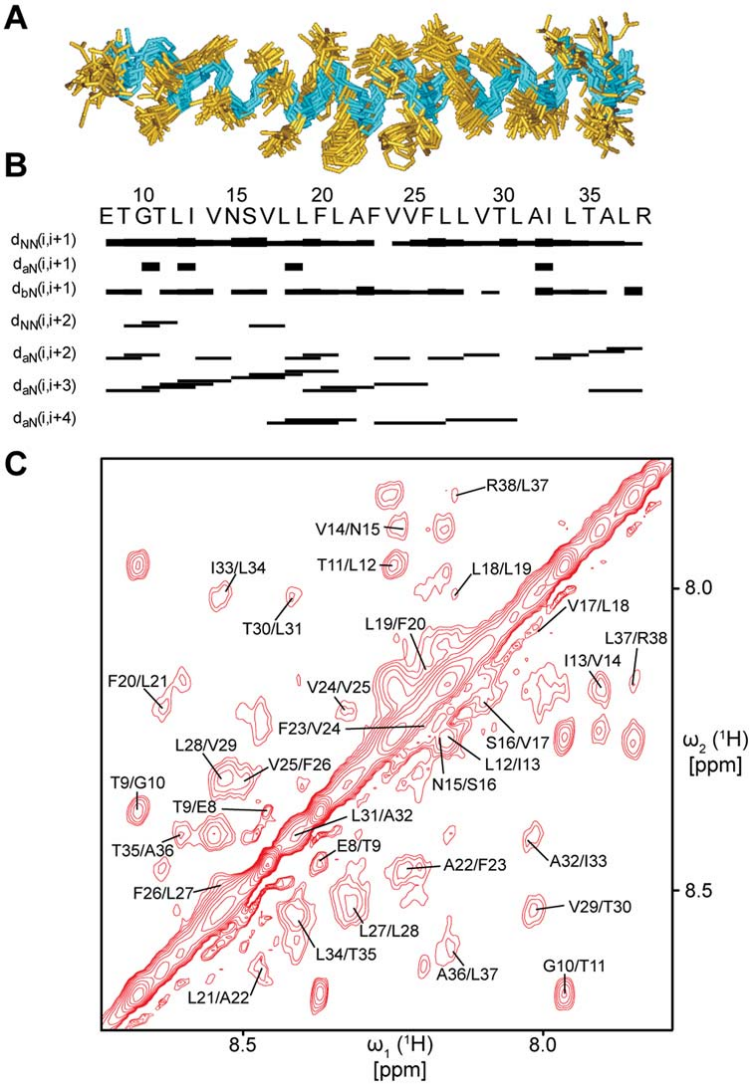
ETM forms a continuous α-helix in DPC micelles. (A) Superimposed 20 conformers of ETM (monomer) calculated from CYANA. The peptide backbone is shown in cyan and the residue side chains in gold. (B) Secondary structure plot for ETM depicted as bands of varying thickness, indicative of the NOE intensity. Sequential and medium range NOE connectivities are shown below the primary sequence. d_NN_ amide backbones and d_αN_(i,i+3), d_αN_(i,i+4) connectivities are mostly continuous throughout the length of the peptide, indicating that the peptides adopt a predominantly α-helical conformation [71]. (C) Representative H^N^/H^N^ region of 2D NOESY spectra.

The 3D structure of the pentameric channel formed by the transmembrane domain of SARS-CoV E (ETM) was reconstructed in several stages (Fig. S1, A–C). In a first stage, the structure of the ETM monomer was calculated using the constraints derived from 492 NOEs. For a set of 20 ETM monomeric conformers, the backbone root-mean-square deviation (RMSD) was less than 1 Å, or 1.5 Å after including side chain heavy atoms (see statistics in Table S1). ETM forms a continuous α-helix encompassing all residues (Fig. 1, A–C), including both N- and C-termini, showing no signs of terminal fraying [39]. Similar results were obtained in the presence of the drugs HMA and amantadine (AMT) (Fig. S2). The latter drug was shown to inhibit in vitro channel activity of a transmembrane domain of SARS-CoV ETM flanked by two N- and C-terminal lysines [37].

**Figure 2:**
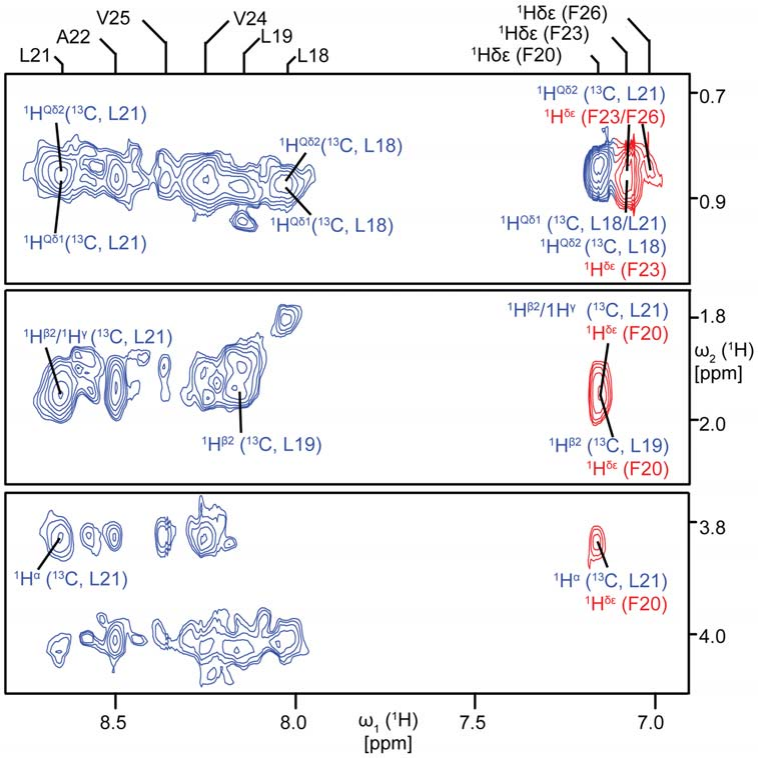
Intra- and inter-monomeric NOEs. Dereference 2D homonuclear ^1^H^N^, ^1^H^aromatic^ band-selected NOESY exhibiting NOEs between ^1^H covalently bound to the ^13^C spins of L18, L19, and L21 and other proximal ^1^H spins. Intramonomeric cross-peaks are shown in blue. Cross-peaks which cannot be explained by intramonomer interactions based on the reconstructed secondary structure are assigned to intermonomer NOEs, and are shown in red.

In a second stage, a representative ETM conformer was selected, and threaded through the pentameric scaffold of ETM [38],[40] while monitoring inter-monomer constraints; out of possible 9 inter-monomer constraints (Fig. 2), only 5 were finally used (Table S2). NOEs were added sequentially, and upon fulfillment of the NOE, the next NOE was added. Ambiguity due to overlap of resonances from ^1^H^β2^ of L19 and ^1^H^β2^/^1^H^γ^ of L21 was resolved by molecular dynamics (MD) and energy minimization to adjust side chain orientations of residues forming the inter-helical interface.

### Orientation of ETM determined using paramagnetic probes and residual dipolar couplings (RDCs)

**Figure 3:**
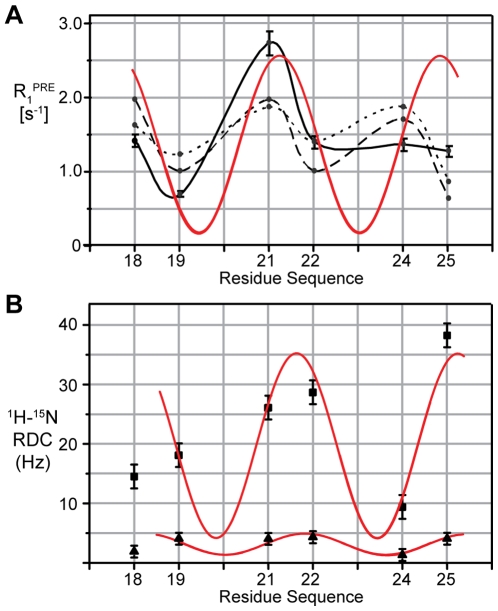
Orientation of ETM relative to the detergent phase and α-helical geometry. (A) PRE rates of ^1^H^N^ nuclei for residues A22, V24, V25, L18, L19 and L21 (black solid line), superimposed to predicted PRE from the ETM pentamer using either the immersion depth method (black dotted line) or the distance from the center method (black dashed line)(Fig. S3, BC). (B) RDCs for ^1^H^N^ nuclei corresponding to A22, V24, V25, L18, L19 and L21 in 4% and 8% polyacrylamide gels compressed axially (▲) or radially (■), respectively. Best-fit sine waves of 3.6 periodicity (red lines) are superimposed onto both PREs and RDCs plots, and show that the stretch of residues 19–24 adopt a regular α-helical structure. The RDCs of flanking residue L18 could not be fitted to this ∼3.6 periodicity, suggesting a local deviation from ideal α-helical geometry.

To validate independently our reconstructed pentameric ETM model, the orientation of the ETM helices relative to the DPC molecules in the micelle was determined using “dipolar waves” [41], i.e., oscillations in the longitudinal relaxation of protons due to the periodically variable proximity of ETM ^1^H^N^ to 16-DSA, a hydrophobic paramagnetic probe confined to the DPC environment. The observed paramagnetic relaxation enhancement (PRE) of the six isotopically labeled residues in ETM (Fig. 3A) was compared with the PRE calculated from our model according to Protocol S1 (and see Fig. S3). The good fit between observed and expected values validates the proposed orientation of the ETM helices in the α-helical bundle. This orientation was further confirmed by the observed broadening of the NOESY crosspeaks from aromatic side-chains of F20, F23 and F26 to aliphatic protons of DPC after addition of 3 mM 16-DSA (not shown). Cross-peaks from ETM N- and C-terminal residues, E8-T11 and T35-R38, remained unaffected, indicating that these residues are exposed to the aqueous environment. Consistent with this, we observed broadening of NOESY cross-peaks from E8-L12 and A36-R38 when 1.5 mM of the water soluble paramagnetic probe gadodiamide was added to a fresh sample (not shown).

Residual dipole couplings (RDCs) were also measured (Fig. 3B) using two different polyacrylamide concentrations and methods of compression. A 4% gel was subjected to axial compression (its lower density allows the application of greater compressive forces) while an 8% gel was subjected to radial compression using a gel press assembly. In both RDC measurements, a sinusoidal wave of residue periodicity of ∼3.6 could be observed from residues 19 to 25, consistent with α-helical periodicity. The RDC of residue L18 could not be fit to this periodicity, due to either deviation from ideal α-helical geometry or to conformational dynamics. The RDCs were used to determine the alignment tensors of the helix, where one of the tensors coincided with the axis of symmetry of the helical bundle, consistent with the helix forming part of an oligomeric complex, as shown previously by other techniques [38]. Thus, to summarize, the present pentameric α-helical bundle model was built using (i) NOE constraints, (ii) paramagnetic relaxation data, (iii) the obtained alignment tensor/axis of symmetry from RDCs, and (iv) the known oligomeric size of the ETM α-helical bundle.

To gain a further insight on the compactness of the channel structure, we monitored the deviation of the observed chemical shifts from those expected in a random coil structure. The periodicity in these chemical shifts was analyzed using wavelets (Fig. S4). For residues 8–18 the periodicity was 2.8 residues per cycle, close to that of a 3_10_ helix (3 residues per turn), for residues 19–30 the periodicity was 6.2, and for residues 32–38 the periodicity was 3.8 residues per cycle, i.e., close to that of a canonical α-helix. We interpret the low periodicity in the central part of the α-helix as due to a tighter packing of the oligomer, i.e., lumenally oriented ETM residues are expected to experience a less hydrophilic environment in this region than in the less compact ends of ETM, leading to a more uniform hydrophobicity around the helix.

**Figure 4:**
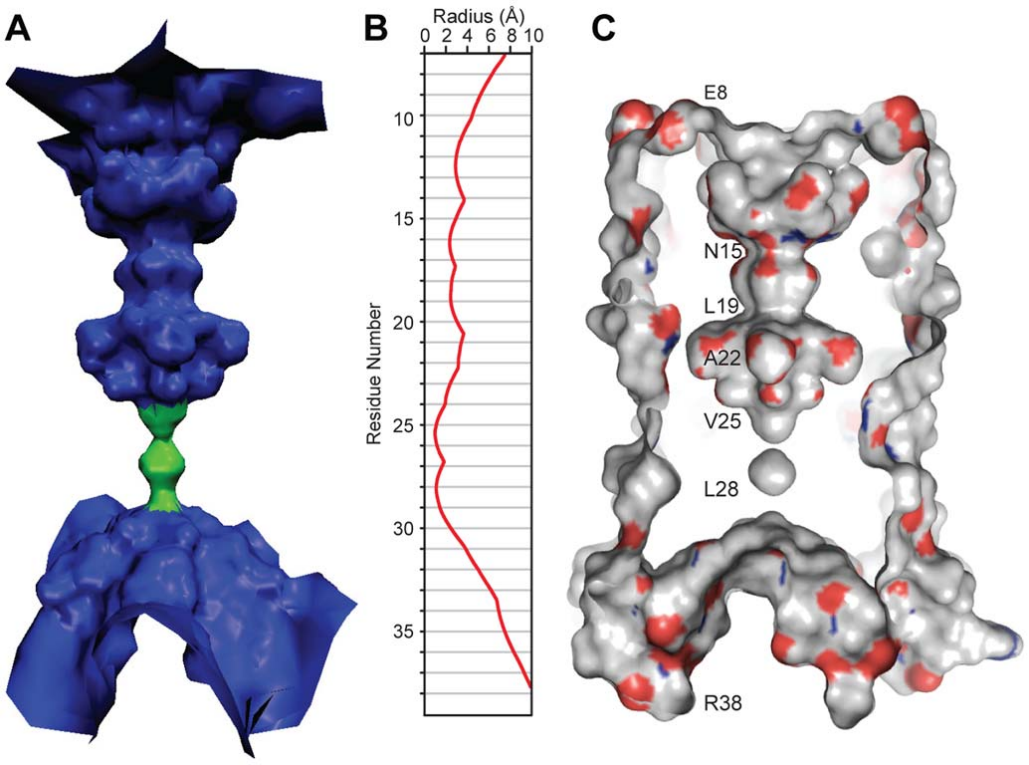
Pentameric structure of ETM. (A) Lumen of the α-helical bundle (blue) formed by the ETM α-helices (omitted for clarity), with most constricted region at V25 (green). (B) Radius of the lumen of the ETM channel as a function of residue number, calculated by HOLE [66]. (C) Side view surface representation of the ETM channel showing the lumen volume. Oxygen and nitrogen atoms are colored red and blue respectively.

The lumen of the pentameric ETM assembly (Fig. 4A) adopts a distinct hour-glass shape. The polar side chains of N15 are oriented towards the lumen and, from the MD simulations, they form a ring with an inner diameter of about 4–5 Å (Fig. 4B, C). The hydrophobic side chains of L18 and A22 line a more spacious region where the diameter reaches ∼7.3 Å. The most constricted part is located between residues V25 and L28 with diameters of 2.0 and 2.3 Å, respectively (Fig. 4B, C).

### Effect of HMA and amiloride on ETM

**Figure 5:**
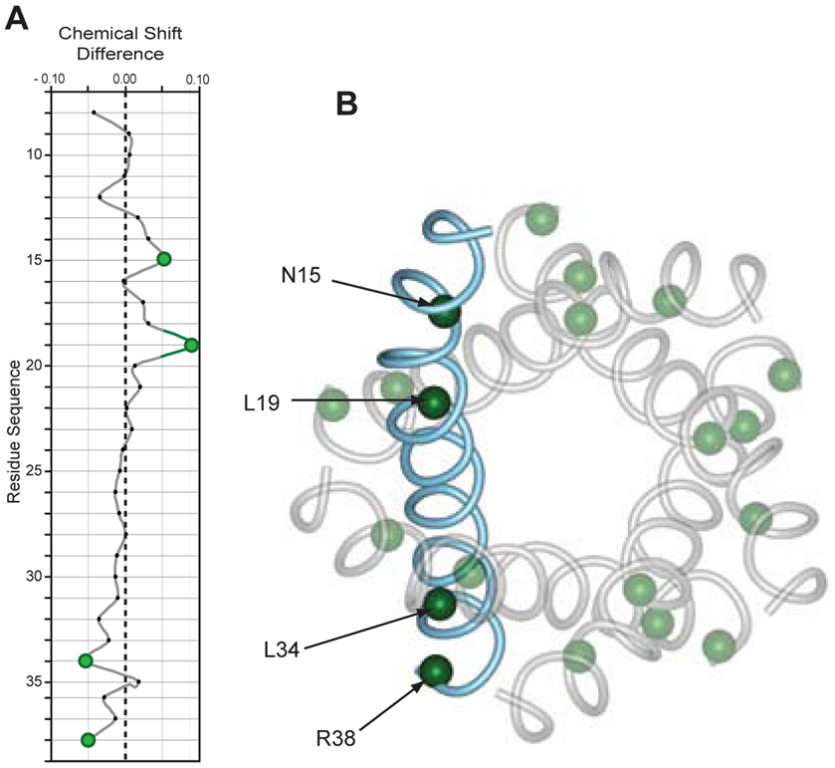
Chemical shift perturbation in ETM induced by HMA. (A) Difference in the ^1^H^N^ chemical shifts (ETM *minus* ETM_HMA_) after addition of HMA at the molar ratio described in Methods. Amides exhibiting deviations of chemical shifts of more than 0.05 ppm are highlighted in green. (B) Top view of the ETM pentameric model showing these amides as small spheres.

It has been reported that the drug HMA, but not amiloride, inhibited in vitro conductance of synthetic MHV E and HCoV-229E E [36], which are close homologs to SARS-CoV E. Therefore, we tested the effects of both drugs on the ETM channel. When ETM in DPC micelles was exposed to HMA, changes in ^1^H^N^ chemical shift were observed throughout the peptide, with most affected ETM amide protons clustering at both ends of ETM, L19 exhibiting the largest chemical shift (Fig. 5A). The NOEs observed between HMA and ETM (Fig. S5) suggest the presence of two binding sites, one near R38 and another near N15. This figure also shows that a protonated form of HMA at nitrogen-5 is bound to the channel. This form may be stabilized by hydrogen bonding to the side-chain carbonyl of N15 and the guanidinium moiety of R38, resulting in an observable ^1^H^N5^ signal at 10.7 ppm (Table S3). In the absence of ETM, this HMA resonance was only observed when the pH was lower than 3.5, indicating a possible role of ETM in the stabilization of this HMA protonated state.

The relative intensities of the cross-peaks assigned to HMA protons indicate that at the N-terminal binding site, near N15, HMA∶ETM stoichiometry approaches 1∶5, i.e., one HMA molecule per ETM pentamer. In contrast, at the C-terminal binding site, near R38, the HMA∶ETM stoichiometry was 1∶2 suggesting for this site a rapid (in the chemical shift time scale) exchange between ETM-bound and micelle-bound forms of HMA. We note that DPC micelles and HMA exhibited identical diffusion rates, indicating that HMA partitions into the detergent phase. The shifts in the [^1^H,^15^N]-HSQC spectrum after addition of HMA are apparent (Fig. S6, AC). Amiloride, in contrast, did not produce significant chemical shift changes (Fig. S6, BD), even at an ETM∶drug molar ratio ten times higher than for HMA (not shown). For comparison, addition of AMT at ten times more concentration than HMA also produced similar chemical shifts as those observed for HMA (not shown). However, in contrast to HMA, no NOEs between AMT and ETM were detected.

It is interesting to note that L19, which was present at a discontinuity point in chemical shift periodicity (Fig. S4, B), also showed a significantly broadened cross-peak in the [^1^H,^15^N]-HSQC spectrum due to conformation exchange processes. By elevating the temperature from 30°C to 37°C, this exchange increased, resulting in sharpening of the L19 cross-peak (Fig. S6, EF). Broadening was also reduced by addition of HMA at 30°C (Fig. S6, G), suggesting stabilization of one of the ETM exchanging conformers by bound HMA. Incidentally, increasing the temperature from 30°C to 37°C also resulted in sharpening of the L18 cross-peak (not shown), indicating that both residues may be involved in a hinge-like motion.

**Figure 6:**
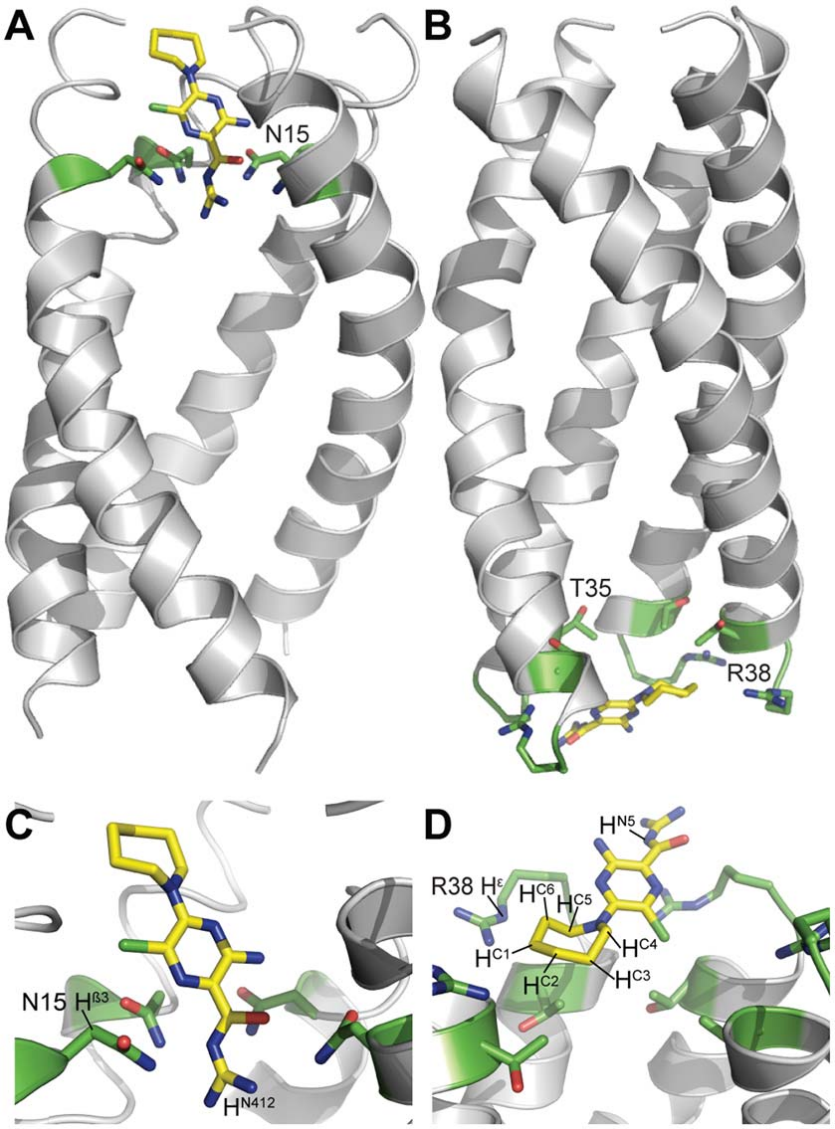
Binding of HMA to the ETM pentameric channel. (A) Side view of the binding of HMA to the ETM pentamer in the vicinity of N15. The side chains of amino acids interacting with HMA are shown using a stick representation. (B) Binding of HMA to the C-terminal binding site of the channel, in the vicinity of T35 and R38. The lowest energy conformation of HMA is shown at the centre of bundle. For clarity, one of the ETM monomers has been removed. (C) and (D), top views of panels (A) and (B), respectively.

The proposed two binding sites of HMA in the ETM channel are shown in Fig. 6. In one binding site, HMA may be stabilized by a hydrogen bonding network to the Asn 15 side chains, with the cyclohexamethylene ring pointing away from the center of the channel (Fig. 6, AC). The second binding location for HMA was observed near the C-terminus of ETM, around residue R38, where the amiloride group of HMA is likely to be involved in interactions with the guanidinium groups of R38 (Fig. 6, BD). The cyclohexamethylene ring was in van der Waals contact with methyl groups of residue T35, i.e., oriented towards the center of the membrane.

### Electrophysiological measurements

**Figure 7:**
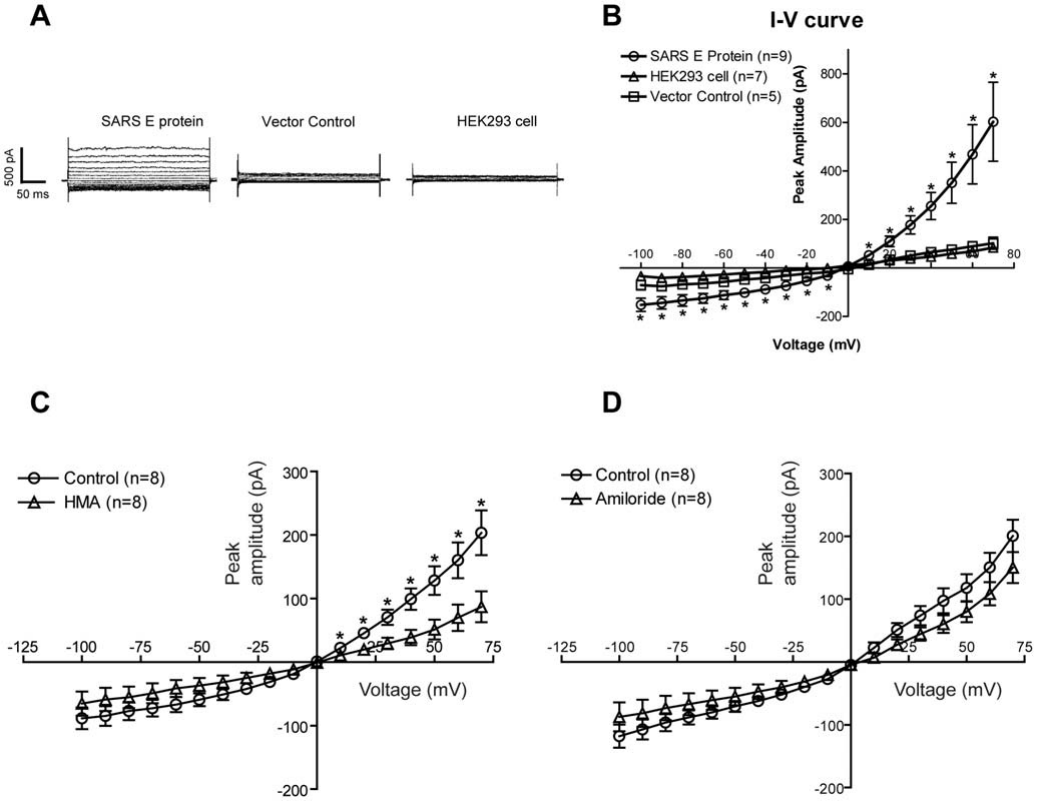
Current-voltage relationship of SARS-CoV E protein and inhibition by HMA. (A) Example traces of current flowing through cells expressing SARS-CoV E protein, vector alone-transfected cells and untransfected HEK-293 cells. The cells were held at 0 mV and stepped to various potentials from −100 to 70 mV (in steps of 10 mV). (B) Whole-cell I–V curve in which peak current amplitudes were plotted against test potentials. Notice the significant large inward and outward currents recorded from SARS-CoV E protein, in contrast to vector alone and untransfected HEK-293 cell controls (*, two-tail unpaired T test, p<0.05, compared to vector control group). (C) HMA (10 µM) significantly reduced the whole cell current through SARS-CoV E protein (*, two-tail unpaired T test, p<0.05, compared to control). (D) same plot for amiloride (10 µM), where the small difference with the control was not found to be statistically significant (see text).

To confirm the relevance of this pentameric structure and the effect of HMA and amiloride on the channel activity, results were obtained by transient expression of SARS-CoV E in human embryonic kidney 293 (HEK-293) cells. Transfected cells produced significantly higher channel activity than the controls (Fig. 7A). The whole-cell patch clamp recording (Fig. 7B) reveal moderate inward (negative current) and large outward (positive current) conductance. The same figure shows significantly smaller ‘control’ currents obtained with cells transfected with the vector alone, or non-transfected HEK-293 cells. ACSF (artificial cerebro-spinal fluid) was used as bath solution, which contained a high concentration of NaCl (124 mM), whereas the internal solution contained a high concentration of potassium ion (145 mM), close to the intracellular medium under physiological conditions. Under our recording conditions, the estimated equilibrium potentials, E_Na_ and E_k_, were 65 mV and −87 mV, respectively. Strong selectivity for either of these cations would produce a reversal potential (i.e., zero current) near their corresponding equilibrium potential. If the channel was poorly selective, the reversal potential would have a value somewhere in between E_Na_ and E_k_, whereas no selectivity would produce a reversal potential in the mid point between these values (∼−10 mV). The observed value of reversal potential at ∼0 mV (Fig. 7B) indicates low selectivity between sodium and potassium, with perhaps a mild preference for sodium. This is consistent with previous results performed in planar lipid bilayers with synthetic E proteins [35],[36].

To test the inhibitory effect of HMA, cells were exposed to 10 µM HMA in the bath solution. This significantly reduced the whole cell current flowing through SARS-CoV E protein; indeed, the mean peak current at 70 mV was reduced by ∼60% (P<0.02, unpaired t-test) (Fig. 7C). Amiloride, in contrast, reduced the mean peak current by only ∼25%, although this difference was not statistically significant (P>0.05, unpaired t-test) (Fig. 7D). In this figure, we note that the peak current for transfected cells recorded in panel B, ∼600 pA, is larger than that in panels C and D, of ∼200 pA. We attribute these differences to variation in cDNA preparation, transfection, and the time of recording following transfection.

## Discussion

### The ETM pentameric channel

ETM shows a sufficiently resolved ^1^H NMR spectra. However, to facilitate resonance assignment and to unequivocally identify inter-monomer NOEs, six labeled amino acids were chemically incorporated near the center of the ETM α-helix. Selection of appropriate specific labels is facilitated by prediction of likely inter-monomer interactions using other lower resolution biophysical techniques. In particular, the model reconstructed here with NMR data is consistent with a model that was derived from the analysis of evolutionary conservation of ETM in coronavirus envelope proteins [40]. The latter approach is data independent, and only relies on the reasonable assumption that all homologues share the same backbone structure [42]. Because by definition conservative mutations that appear during evolution should not destabilize the correct model of transmembrane interaction, but may destabilize incorrect low energy models that appear during the simulations along with the correct model, these mutations act effectively as an *in silico* filter [43]. The inter-helical orientation obtained for the ETM α-helices, and their orientation respect to the lumen of the ETM channel and detergent phase, is also in agreement with previous ETM helix rotational orientation measurements obtained by infrared linear dichroism [38].

Our model shows a 2–2.3 Å wide constriction formed by the side-chains of V25 and V28. This is probably not wide enough for the passage of sodium ions, which suggests this represents a closed state of the ETM channel. The ^1^H-^15^N dipolar couplings from the six labeled backbone amides exhibited a periodicity of 3.6, consistent with a canonical α-helical periodicity, except for L18 which was found to be an outlier, i.e., its ^1^H-^15^N vector points in a direction not consistent with the other labeled residues. Additionally, the amide groups of L18 and L19 showed significant line broadening, which was reduced at more elevated temperature likely due to acceleration of the exchange rates. We interpret this as a conformational exchange-induced transverse relaxation at these residues, and we speculate that these conformational dynamics may be required for the channel's function. Similar band narrowing was observed after addition of the drug HMA (see below).

### Ion selectivity of ETM

In a previous report [36], it was suggested that synthetic CoV E proteins have cation selective channel activity, with selectivity (P_Na_/P_K_) of 0.25 for HCoV-229E, 69 for MHV E, 10 for SARS-CoV E and 3 for IBV. In the present work, we observed a very mild preference for sodium over potassium. According to these data, only the apparent selectivity of HMV for sodium appears to be significant. The diameter of naked Na^+^ is around 2 Å, and that of K^+^ is 2.66 Å, and the diameter of the ETM pore at the level of N15 (4 to 5 Å) is sufficient to accommodate a single dehydrated Na^+^ or K^+^ ion. Hence it may be speculated that N15, or its polar equivalent in other sequences, form a selectivity filter for cations. The equivalent residue to SARS-CoV E N15 in MHV E is Gln (Fig. S7), which has a one methylene longer side chain. This may lead to further occlusion of the channel at this position, and may explain the observed higher selectivity for sodium in MHV E. We also note that the lumen-facing orientation of Asn and Gln may also have a structural role, as these residues are known to stabilize transmembrane interactions [44]–[46].

### Inhibition by HMA and binding site in ETM

In the present work, we localized two binding sites for HMA. We speculate that the localization of HMA near N15 could be similar in other CoV E proteins because this position (lumen-exposed) is always occupied by a polar residue in other CoV E sequences (N, Q, S, T) (Fig. S7). However, HMA sensitivity has only been shown for E proteins that contain a long polar side chain at this position, e.g., N (SARS-CoV E (this paper), HCoV-229E [36]) or Q (MHV E [36]); IBV E, which contains a smaller polar side chain (Thr) was HMA-insensitive [36]. It would be interesting to test if E proteins containing a small polar side chain at this position, e.g., S or T, are generally HMA insensitive.

Similarly, at the C-terminal end of ETM, in the position equivalent to R38 in SARS-CoV E, a basic residue is often found in other E sequences (Fig. S7). Additionally, in HMA-sensitive CoV E proteins, at least one of the lumen-facing residues immediately below R38 is polar: TA in SARS-CoV E, AS in MHV E, and KL in HCoV-229E. For IBV E (group 3) which was reported to be HMA insensitive [36] there is no polar residue at this position (AF pair). We note, however, that R38 is the C-terminal residue in ETM, and may not be involved in HMA binding in the context of the full length protein. Experiments to clarify this point using an extended ETM or full length SARS-CoV E are in progress.

For cells infected with MHV, the EC_50_ for HMA was ∼4 µM, whereas in an E-deleted virus (MHVΔE), no effect was observed after HMA addition, pointing to E protein as the HMA target [36]. HMA also inhibited HCoV-229E replication in cultured cells, with an EC_50_ of ∼1 µM. In neither case, however, did amiloride have antiviral activity on replication in cultured cells. Consistent with these results, we show that channel activity of transfected mammalian cells expressing SARS-CoV E is inhibited by extracellular HMA, but not by amiloride, suggesting a specific activity.

SARS-CoV E in plasma membranes is oriented with the N-terminus facing the cytoplasm [47], whereas the C-terminus of the ETM would face the extracellular domain. The latter therefore would be the likely HMA binding site in our patch clamp experiment, although the fact that HMA partitions into detergent micelles, and presumably into lipid bilayers, suggests that both N and C-termini of ETM could be accessible to the drug.

The weak inhibition observed for amiloride is consistent with our NMR data, because addition of amiloride to ETM showed an increase in line broadening, but only small changes in peak positions, suggesting a global perturbation of protein structure but not a specific interaction. Finally, although the chemical shifts induced by AMT (not shown) were similar to those observed for HMA, we did not observe NOEs between AMT and ETM. This is not unexpected; in contrast with what we observed in a lysine-flanked ETM peptide [37],[38], the in vitro ion channel activity observed for ETM without flanking lysines, i.e., like the one used herein, was not inhibited by AMT [38].

The flexibility encountered around residues 18–19, which was reduced by temperature or by addition of HMA, is reminiscent of the changes observed in the influenza A channel M2 after addition of AMT. The M2 open state (low pH) has been shown to be dynamic or heterogeneous [48], as opposed to the less flexible closed state (high pH). Addition of AMT to M2 caused substantial narrowing of ^15^N spectra [48] and a reduced M2 conformational distribution in a MAS ^13^C and ^15^N NMR study [49], both indicative of a more rigid M2 channel in the presence of the drug. The latter studies conform to a model where M2 accesses several conformational states, and AMT would stabilize a ‘closed’ conformation. In SARS-CoV E, a similar rigidization of ETM may be partly responsible for inhibition, although physical blockage to ion passage is also possible. A more complete ETM labeling approach, which is in progress, will undoubtedly shed more light on the nature of this inhibition.

### Effect of extramembrane domain of SARS-CoV E on stability

Another important issue is the effect of the extramembrane domain on channel function and stability. For example, in M2 proton channel from influenza A, truncating the cytoplasmic tail alters ion channel activity when M2 is expressed in oocytes of Xenopus laevis [50], and analytical ultracentrifugation showed that the full-length protein stabilizes the M2 tetramer by approximately 7 kcal/mol [51]. In SARS-CoV E, preliminary sedimentation equilibrium experiments (unpublished data) suggest a slightly lower association constant in a monomer-pentamer equilibrium for full length SARS-CoV E protein, or for a synthetic ETM spanning residues 7–42 (K_a_∼10^15^), when compared to ETM_8–38_ (K_a_∼10^17^) [38]. Thus, the extramembrane residues may be slightly destabilizing for the SARS-CoV E pentamer.

### Conclusion and final remarks

In recent years, several viral proteins have shown membrane permeabilization properties or ion channel activity, e.g., poliovirus 2B, alphavirus 6 K, HIV-1 Vpu, and influenza virus M2, and have been named collectively as ‘viroporins’ [52]. However, the physiological relevance of this activity has only been shown conclusively for M2, a well known pharmacological target that is inhibited by AMT for which a detailed structure is available [53]–[55]. Electrophysiological data, as well as detailed structural information is lacking for most of these proteins. We show in the present work that SARS-CoV E possesses channel activity not only in vitro, but also when expressed in mammalian cells, and we have structurally characterized the homo-pentameric transmembrane domain (ETM) responsible for this activity, when solubilized in DPC micelles in the absence or presence of small drugs.

Although the precise role of this proposed channel activity is not known, it is possible that it leads to a subversion of ion homeostasis in the host cell that could account for the observed attenuation of E-deleted coronaviruses (see above). For example, in hepatitis B virus, calcium homeostasis regulation by HBx protein has been shown to be essential for replication [56]. It is also possible that the observed pro-apoptotic effect of SARS-CoV E protein in T cells [57] could be mediated by a disruption of cell ion homeostasis and membrane depolarization, a general marker of apoptosis [58].

## Methods

### Peptide synthesis

Isotopically labeled amino acids were derivatized with 9-fluorenyl-methyloxycarbonyl (FMOC) [59]. The ETM peptide, corresponding to the transmembrane domain of SARS-CoV E (residues 8–38), E_8_TGTLIVNSVLLFLAFVVFLLVTLAILTALR-NH_2_, was synthesized using standard solid phase FMOC chemistry (Intavis Respep peptide synthesizer). The peptide was cleaved from the resin with trifluoroacetic acid (TFA). The lyophilized peptide was purified by HPLC, as described previously [21]. Lyophilization was performed in the presence of HCl (at the molar ratio of 20∶1, HCl∶peptide) in order to avoid formation of peptide-TFA adducts; consequently, the TFA band at ∼1685 cm^−1^ was absent in the infrared amide I region (not shown). Peptide purity was further confirmed by electrospray ionization (ESI) mass spectrometry. During ETM synthesis, ^15^N-labeled amino acids were introduced at positions A22, V24, V25, and ^13^C,^15^N-labeled amino acids at positions L18, L19 and L21.

### NMR sample preparation

Approximately 1.6 mg of lyophilized ETM peptide was solubilized in phosphate-buffered saline (PBS, 10 mM Na_2_HPO_4_·NaH_2_PO_4_) containing 17 mg of DPC (Avanti Polar Lipids), to a molar ratio of 1∶100 (peptide∶DPC). Under these conditions, sedimentation equilibrium studies have shown ETM to be pentameric [38]. For AMT binding experiments, the NMR sample was titrated stepwise with AMT (1 amino-adamantane) hydrochloride powder (Fluka) dissolved in PBS, pH 5.5, up to a final molar ratio of 1∶100∶100 (peptide∶DPC∶AMT). For HMA (5-N,N-Hexamethylene amiloride, Sigma) and amiloride (amiloride hydrochloride, Sigma) binding experiments, aliquots of HMA (solubilized in D_6_-DMSO, Cambridge Isotopes) or amiloride (solubilized in water) were added to an empty NMR tube. In both cases, solvent was removed by lyophilization followed by addition of ETM/DPC solution to a molar ratio of 1∶100∶10 (peptide∶DPC∶drug), i.e., ten times less than for AMT (see above). The resulting mixture was heated to 40°C for 30 min, vortexed and equilibrated at 30°C for a few hours before collecting NMR spectra.

For the sample preparation in the presence of 16-doxyl stearic acid (16-DSA), the desired amount of 16-DSA was first dissolved in methanol. The aliquots of 16-DSA corresponding to 1 mM, 3 mM and 5 mM of 16-DSA in final NMR samples were added to an empty NMR tube and dried under a stream of dry N_2_ gas. The NMR sample containing ETM/DPC was added to the NMR tube containing the dry 16-DSA and was left to equilibrate for a few hours. Gadodiamide (OMNISCAN; gadolinium chelated with 2-[bis[2-[(2-methylamino-2-oxoethyl)-(2-oxido-2-oxoethyl)amino]ethyl]amino]acetate, GE Healthcare) was used from a 0.5 M stock solution and was diluted to 1.5 mM.

Weakly aligned samples were prepared by soaking a 1 mM solution of selectively labeled ETM in 100 mM DPC into polyacrylamide gels. Two different acrylamide concentrations, 4% and 8%, at axial and radial compression, respectively, were used to independently verify the experimental results. Gels were prepared from stock containing 36% w/v acrylamide (Bio-Rad Laboratories) and 0.94 w/v N, N-methylenebisacrylamide (Bio-Rad Laboratories) which yields an acrylamide/bisacrylamide molar ratio of 83∶1.4% acrylamide gels were cast in 4.2 mm inner diameter (ID) glass tubes, while 8% gels were cast in a gel chamber of 5.4 mm ID (New Era Enterprise, Inc). After complete polymerization, gels were washed in large excess of H_2_O overnight to ensure removal of un-reacted components. Gels were then dried to completeness at 37°C. Peptide solutions were soaked into the dried gels overnight to ensure complete re-hydration. The 4% gel was carefully added into a 4.2 mm ID Shigemi tube (Shigemi Co. Ltd.) and compressed axially using the supplied Shigemi plunger; the 8% gel was radially compressed into a 4.2 mm ID open-ended tube using the gel press assembly (New Era Enterprise, Inc), and secured using the supplied support rod and end gel plug.

### NMR spectroscopy

NMR experiments were performed at 30°C using Bruker Avance-II 700 and 600 NMR spectrometers equipped with cryogenic probes (Bruker BioSpin). Complete sequence-specific assignment of backbone ^1^H^N^ was achieved using 2D homonuclear ^1^H^N^, ^1^H^aromatic^ band-selected NOESY (Fig. S8), 3D ^15^N resolved NOESY-HSQC, 3D ^13^C resolved NOESY-HSQC and 3D ^15^N HSQC-NOESY. Intra-monomer NOEs involving both backbone and side-chain protons were assigned using the same set of 2D and 3D NOESY spectra. Mixing time for all NOESY spectra was set to 200 ms.

To identify inter-monomer contacts, we constructed a difference between two 2D ^1^H^N^, ^1^H^aromatic^ band-selected NOESY spectra, acquired with and without ^13^C decoupling during the t_1_ chemical shift evolution period. Based on the reconstructed secondary structure of ETM, NOEs were identified as inter-monomeric, i.e., between ^1^H covalently bound to L18, L19 or L21 and other proximal ^1^H spins, if they could not be explained by intra-monomer distances. The amplitudes of inter-monomer NOEs were used to define the corresponding upper limit constraints. Two sets of HMA ^1^H resonances were assigned using 2D TOCSY and 2D NOESY spectra. NOEs between ETM and HMA were identified by direct comparison of NOESY spectra, measured with and without the presence of the drug (Fig. S5).

The orientation of the ETM α-helices with respect to the lipid hydrocarbon phase was verified by the paramagnetic enhancement induced by 16-DSA in the longitudinal ^1^H^N^ relaxation of the six labeled amino acids. The saturation recovery method in a series of [^1^H,^15^N]-HSQC experiments with a variable inter-scan delay was employed. Using two different approaches, the experimental data obtained was compared to the expected paramagnetic relaxation enhancement (PRE) from our proposed pentameric structure (Protocol S1 and Fig. S3). DSS (sodium 2, 2-dimethyl-2-silapentane-5-sulfonate) was used as the internal reference for ^1^H nuclei. The chemical shifts of ^13^C and ^15^N nuclei were calculated from the ^1^H chemical shifts [60]. ^1^H-^15^N residual dipolar couplings (RDCs) were determined using TROSY-anti-TROSY spectra. The acquired data was analyzed with MODULE [61].

### Structure reconstruction

The structure of the ETM monomer in DPC micelles was calculated using the site-specific assignment of ^1^H, ^13^C and ^15^N resonances and unassigned NOEs as input for the program CYANA [62],[63]. Structure calculations started from 100 random conformers, using the standard simulated annealing protocol in CYANA. The statistics of meaningful NOE distance constraints in the final CYANA cycle (Table S1) showed a high density of structural constraints per amino acid. Seven cycles of NOE assignment and structure reconstruction resulted in a bundle of 20 conformers with the average target function values below 0.15.

A symmetrical ETM homo-pentameric structure was reconstructed (Fig. S1, A–C) starting with a backbone model based on orientational data from site-specific infrared dichroism (SSID), which defined helix tilt and rotational pitch angles for residues L21, A22, F23, and V24 [38]. The ETM α-helix built from NMR data was superimposed onto the pentamer skeleton to obtain the full atomic description of the model. This model was subjected to energy minimization to resolve steric clashes, following which, inter-helical NOEs (Table S2) were used as constraints in molecular dynamics (MD) simulations to refine the structure. Inter-helical NOE constraints were applied one by one; only when the system reached equilibrium, another constraint was added. Upon inclusion of all inter-helical constraints, the refined final model was compatible with both site specific infrared dichroism and NMR data.

The energy minimization and all the restrained MD simulations were performed using GROMACS [64] at an atomistic level of detail, using the OPLS-AA [65] force field. Atomic charges were assigned on the basis of the default atomic charge values specified in the OPLS-AA force field. The Van der Waals interactions were modeled using a cut-off distance of 9.0 Å. In the simulation, the cell temperature was maintained at 298.15 K using the Berendsen temperature coupling algorithm. The Berendsen pressure coupling algorithm was applied to maintain the pressure of 1.0 bar. With backbone positions restrained, each inter-helical distance constraint includes a 500 ps simulation, enough for the side chains to move into a conformation that is constraint allowed. The lumenal dimensions for the pentameric model were calculated using HOLE [66] and were visualized using VMD [67].

### Docking of AMT and HMA

According to the chemical shift changes observed after addition of HMA, two ETM pentameric models were obtained. For one model, residues 8–12 and 17–38 did not change after exposure to HMA. For the second model, residues 8–30 did not change. Docking of HMA to these two models was performed using Glide [68],[69] with standard parameters, guided by NOE constraints, and allowing for HMA flexibility. The binding site was defined in terms of two concentric cubes: the bounding box, which contains the center of any acceptable ligand pose, and the enclosing box, which contains all ligand atoms of an acceptable pose. Upon completion of each docking calculation, the best docked structure was chosen using a Glidescore (Gscore) function, a modified and extended version of the empirically based Chemscore function.

### SARS-CoV E construct and transient expression of SARS-CoV E cDNA in HEK-293 cells

The full-length SARS-CoV E protein gene was cloned into pIRES-AcGFP1 (Clonetech) vector by using the restriction enzymes BglII and PstI. The identity of the insert was confirmed by DNA sequencing. In a 35 mm Petri dish, 1.65 µg of human SARS-CoV E cDNA was transiently transfected into HEK-293 cells using the standard calcium phosphate method [70]. The vector pIRES-AcGFP1 was also transiently transfected in separate experiments as a control. Another control was the use of untransfected HEK-293 cells. The cells were grown for 36–48 h in a 5% CO_2_ incubator at 37°C before whole-cell patch clamp recordings.

### Electrophysiological recordings and data analysis

Whole-cell current was recorded at room temperature using the standard patch clamp technique, 48–72 h after transfection. The bath solution contained the following (mM): 124.0 NaCl, 3.5 KCl, 1.0 NaH_2_PO4, 26.2 NaHCO3, 1.3 MgSO4, 2.5 CaCl_2_ and 10.0 D (+)-glucose; gassed with a mixture of 95% O_2_ and 5% CO_2_; pH 7.4, and an osmolarity of 300 mOsmkg^−1^. The internal solution (pipette solution) contained the following (mM): 135.0 potassium gluconate, 10.0 KCl, 10.0 Hepes buffer, 0.5 EGTA, 2 Mg-ATP (pH adjusted to 7.3 with KOH; osmolality 275–285 mOsmkg^−1^). The voltages were uncorrected for a −9 mV junction potential, and actual voltage is obtained by subtracting 9 mV from the reported values. Whole-cell currents, obtained under voltage clamp with an Axopatch Multiclamp700B amplifier (Axon Instruments), were filtered at 1–5 kHz and sampled at 5–50 kHz. The access resistance R_a_ (usually less than 20 MΩ) and the capacitive transients were not compensated. Stock solutions of amiloride and HMA (Sigma) at 100 mM were prepared in 50% DMSO∶50% methanol. To determine if the amiloride derivatives blocked SARS-CoV E protein ion channel conductance in HEK-293 cells, after ion channel currents were detected, 10 µM of the drug diluted in bath solution was applied to the cell.

### Accession numbers

The accession numbers for the proteins in this paper are SARS-CoV E, NP_828854; TGEV E, AAZ91440; HCoV-229E E, NP_073554; MHV E, O72007 and IBV E, P05139.

## Supporting Information

Figure S1 Building the ETM α-helical pentameric bundle. The skeleton of the ETM bundle (A) was based on orientational data from site specific infrared dichroism [38]. The ETM monomer built from NMR data was superimposed onto the skeleton (B) to obtain the full atom description of the model (C). (D) Contact between L18 ^1^H_3_^δ1^ and F23 ^1^H^δε^ (6.03 Å) using the initial ETM helix and (E) after refinement (3.86 Å).
(3.61 MB TIF)

Figure S2 (A–B) Same as Fig. 1 (A–B), for ETM in the presence of 10 mM HMA. The 2D NOESY spectrum is not shown due to large interference from HMA, causing spectral overlap. (C–E) Same as Fig. 1 (A–C) for ETM in the presence of 100 mM AMT.
(5.98 MB TIF)

Figure S3 (A) Schematic representation of the ETM channel surrounded by a DPC micelle that incorporates the paramagnetic probe 16-DSA. The ratio a, between the volume accessible by the probe (red) and the total volume occupied by the micelle (blue), and the distances R_b_ and R are indicated. (B) Schematic representation of the “distance to the surface” model for the calculation of PRE. (C) The same for the “distance from the center” model (see Protocol S1 for details). (D) Structure and characteristic dimensions (in Å) of the doxyl paramagnetic moiety of 16-DSA, with the unpaired electron on the oxygen atom indicated by a dot.
(7.61 MB TIF)

Figure S4 (A) Deviation of ^1^H^N^ chemical shifts from random coil values in ETM (black trace). (B) Wavelet analysis of ETM amide chemical shifts shown in (A) yielded three types of periodicities as indicated by red/orange regions: the N-terminal, central and C-terminal regions showed periodicities (1/n) of 1/2.8, 1/6.2 and 1/3.8 residues, where number n indicates the number of residues required to complete a cycle in chemical shift variation. In (A), the red trace shows the of 100 mM AMT; the data for 10 mM HMA was similar (not shown).
(8.21 MB TIF)

Figure S5 (A) 2D ^1^H^N^, ^1^H^aromatic^ band-selected NOESY spectra of ETM in the presence of 10 mM HMA. The assignment of ETM and HMA resonances, as well as NOEs between the protein and HMA (blue lines) are indicated. (B) HMA molecule, with atoms numbered as in the Protein Data Bank (PDB) structure. (C) 2D ^1^H^N^, ^1^H^aromatic^ band-selected NOESY and 2D water gate NOESY spectra of ETM in the absence (magenta) and presence (black) of 10 mM HMA. The assignment of two sets of connected spin systems from HMA bound at the N- and C-termini of ETM are indicated by green and blue lines, respectively. The assignment of selected NOEs is indicated.
(3.14 MB TIF)

Figure S6 [^1^H,^15^N]-TROSY of ETM in DPC titrated at the final concentrations indicated (inserts) with (A) HMA at 30°C (B) amiloride at 37°C. (C) Same as in (A), but showing only two conditions: no drug and highest drug concentration tested. In (A) and (C), the shift for L19 is indicated by an arrow. (D) Same as (C), but for amiloride instead of HMA. No significant change in peak position is seen for amiloride at the highest concentration tested. (E) A fragment of the [^1^H,^15^N]-TROSY of ETM in DPC, comprising cross-peaks of Leu 19, Val 24 and Val 25, measured at 30°C; (F) the same at 37°C; (G) the same but in the presence of HMA, at 30°C. Results using AMT were similar to those with HMA (not shown).
(1.80 MB TIF)

Figure S7 Clustal X sequence alignment of envelope proteins in coronaviruses, up to the totally conserved Pro residue (P54 in SARS-CoV E), corresponding to SARSCoV sequences (group 2b, black), group 1 sequences (blue), group 2 sequences (red) and group 3 sequences (green). The accession numbers are indicated next to the common name, on the left. The positions N15 and R38 in the SARS-CoV E sequence are indicated above, and the residues that are exposed to the lumen of the pore in ETM are shown with a yellow background. The locations of the two HMA binding sites in the ETM channel are indicated by a red arrow.
(1.86 MB TIF)

Figure S8 Scheme of 2D ^1^H^N^, ^1^H^aromatic^ band-selected NOESY, an experiment suitable for detection of ^1^H^N^, ^1^H^aromatic^ resonances in membrane proteins in the presence of strong aliphatic resonances of solubilizing detergents. Longitudinal relaxation acceleration scheme [72] prevents saturation of longitudinal magnetization of aliphatic spins and water building up during the mixing period τ_mix_. This magnetization is used to accelerate relaxation of amide and aromatic protons to steady-state Boltzmann thermal equilibrium during the inter-scan delay *d*_1_. The radiofrequency pulses on ^1^H, ^15^N, ^13^C are applied at 4.7, 118 and 40 ppm, respectively. Narrow and wide black bars indicate non-selective π/2 and π rf-pulses applied with the phase x unless indicated otherwise. Complex shapes on the line marked ^1^H indicate the ^1^H^N^, ^1^H^aromatic^ band-selective 1.5 ms excitation E-Burp2 pulses with the phase ϕ_3_ and ϕ_4_ and γ*B_1_* = 2733 Hz and the 1.8 ms refocussing Re-Burp pulse [73] with the phase ϕ_4_ and γ*B_1_* = 3050 Hz. The center of the excitation of all ^1^H^N^, ^1^H^aromatic^ bandselective pulses is placed at 8.5 ppm. The durations and strengths of the pulsed magnetic field gradients (PFG) applied along the z-axis are selected as G_1_: 500 µs, 80 G/cm; G_2_: 900 µs, 60 G/cm; G_3_: 900 µs, 70 G/cm. Two datasets, with and without ^13^C-composite inversion pulse decoupling pulse are acquired using the phases ϕ_1_ = x; ϕ_2_ = x; ϕ_3_ = x, y); ϕ_*rec*_ = (x, −x). The quadrature detection in *t*_1_ dimension is achieved by the States-TPPI method [74] applied to ϕ_1_. Subtraction of the datasets results in a 2D NOESY spectrum containing NOEs stemming from ^1^H covalently bound to ^13^C spins and the other proximal ^1^H spins.
(0.66 MB TIF)

Table S1 Statistics of ETM structure reconstruction, alone or in the presence of amantadine and HMA.
(0.06 MB DOC)

Table S2 Inter-helical NOEs for ETM derived from difference 2D homonuclear ^1^H^N^, ^1^H^aromatic^ band-selected NOESY.
(0.04 MB DOC)

Table S3 Assignment of two forms of HMA bound to the N- and C-termini of ETM.
(0.04 MB DOC)

Protocol S1 Supplementary methods.
(0.02 MB PDF)
