# Supplementary figures and images for: Structure and Inhibition of the SARS Coronavirus Envelope Protein Ion Channel
Source: PLoS Pathog. 2009 Jul 10;5(7):e1000511. doi: 10.1371/journal.ppat.1000511 (PMC2702000; doi:10.1371/journal.ppat.1000511)

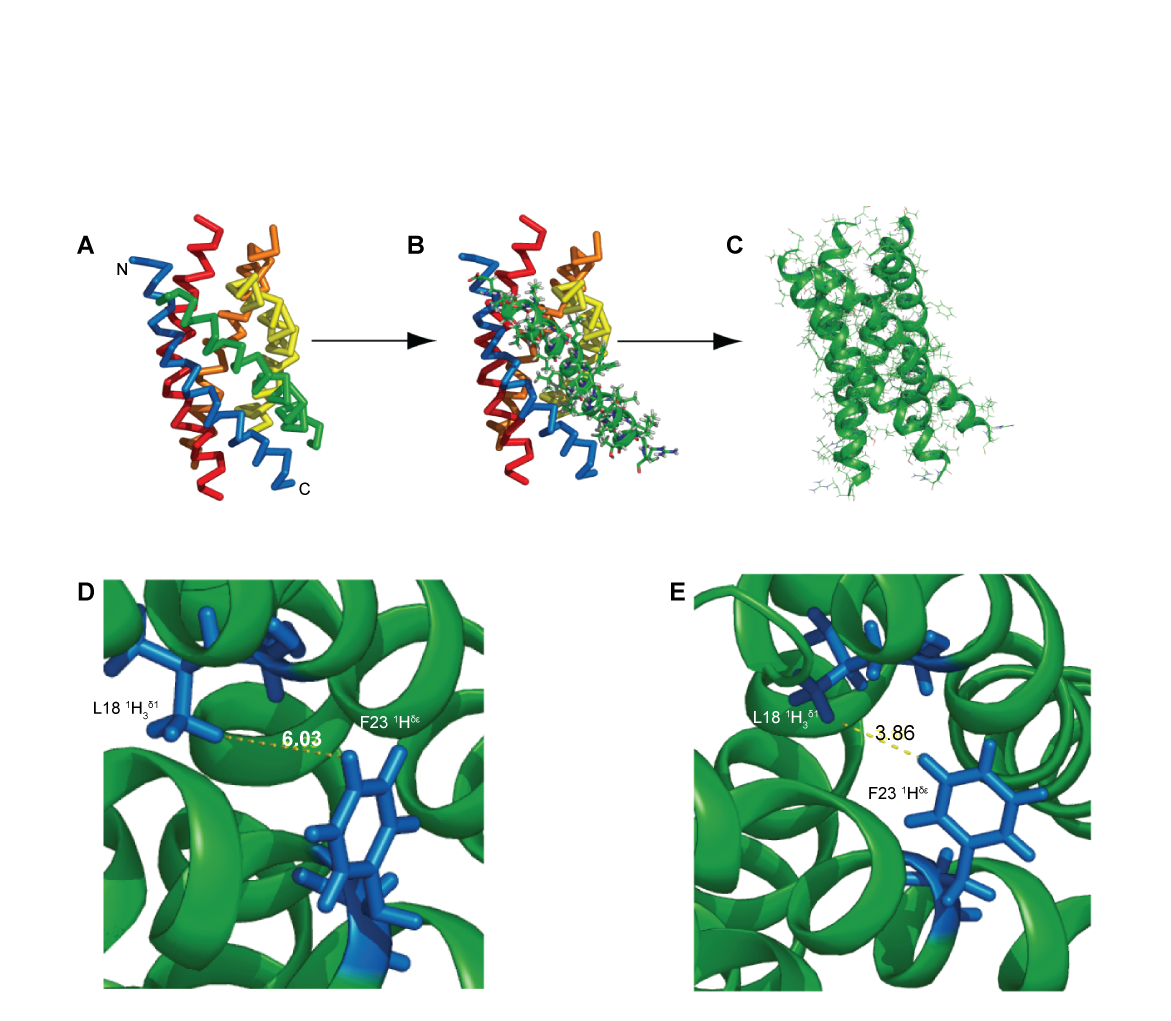

Supplement: Figure S1 — Building the ETM α-helical pentameric bundle. The skeleton of the ETM bundle (A) was based on orientational data from site specific infrared dichroism [38]. The ETM monomer built from NMR data was superimposed onto the skeleton (B) to obtain the full atom description of the model (C). (D) Contact between L18 1H3 δ1 and F23 1Hδε (6.03 Å) using the initial ETM helix and (E) after refinement (3.86 Å). (3.61 MB TIF) [file ppat.1000511.s001.tif]

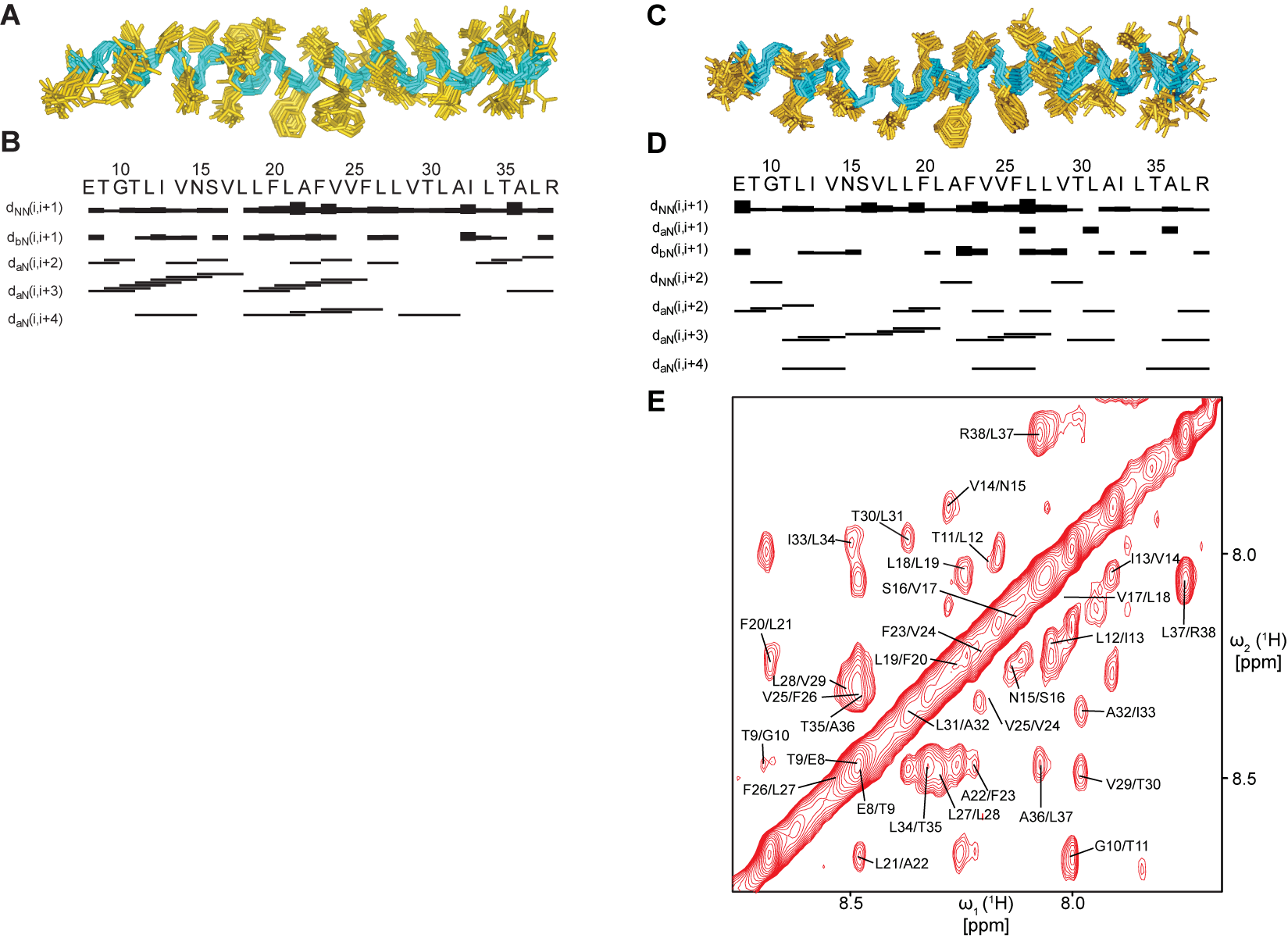

Supplement: Figure S2 — (A–B) Same as Fig. 1 (A–B), for ETM in the presence of 10 mM HMA. The 2D NOESY spectrum is not shown due to large interference from HMA, causing spectral overlap. (C–E) Same as Fig. 1 (A–C) for ETM in the presence of 100 mM AMT. (5.98 MB TIF) [file ppat.1000511.s002.tif]

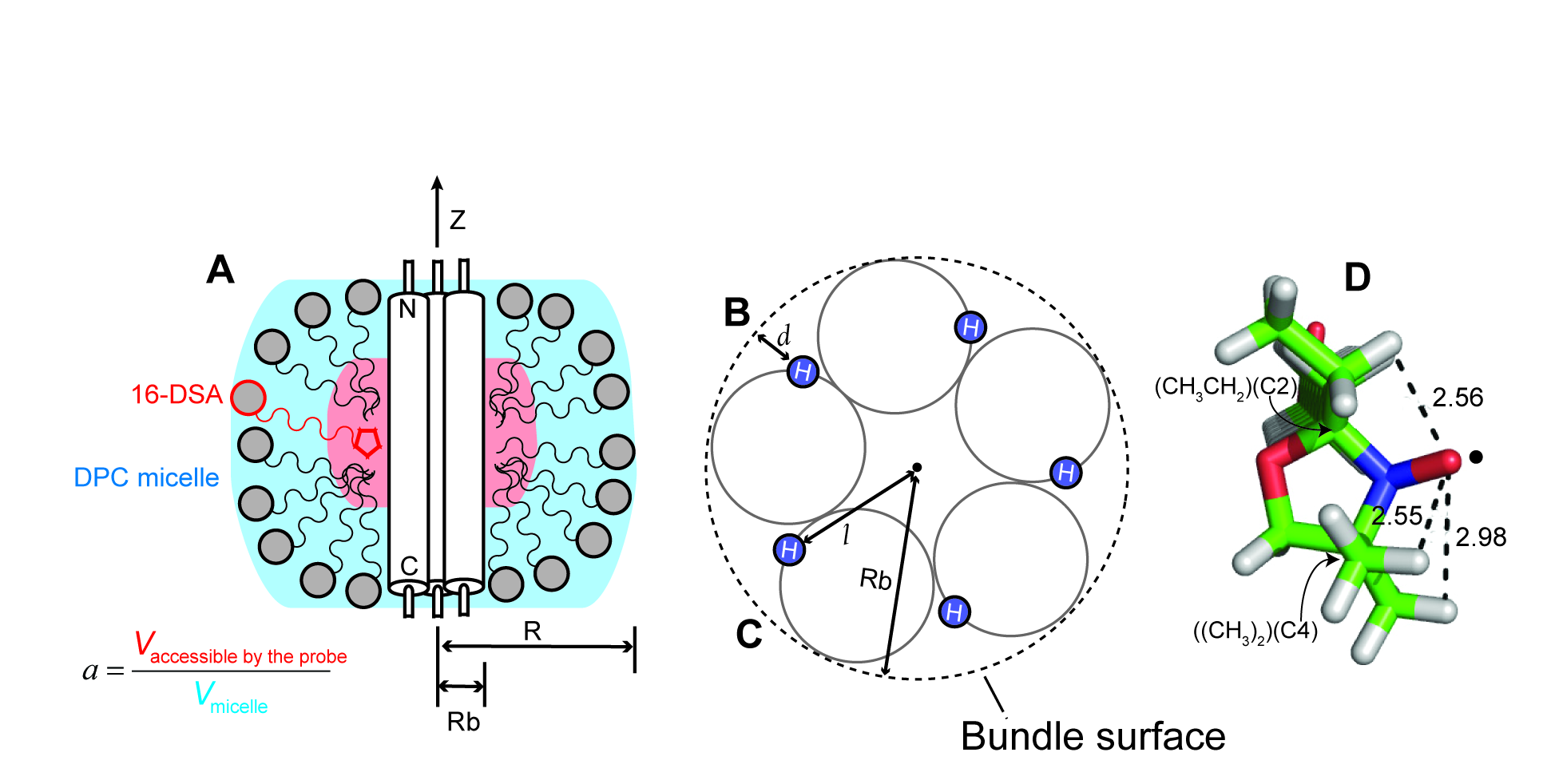

Supplement: Figure S3 — (A) Schematic representation of the ETM channel surrounded by a DPC micelle that incorporates the paramagnetic probe 16-DSA. The ratio a, between the volume accessible by the probe (red) and the total volume occupied by the micelle (blue), and the distances Rb and R are indicated. (B) Schematic representation of the “distance to the surface” model for the calculation of PRE. (C) The same for the “distance from the center” model (see Protocol S1 for details). (D) Structure and characteristic dimensions (in Å) of the doxyl paramagnetic moiety of 16-DSA, with the unpaired electron on the oxygen atom indicated by a dot. (7.61 MB TIF) [file ppat.1000511.s003.tif]

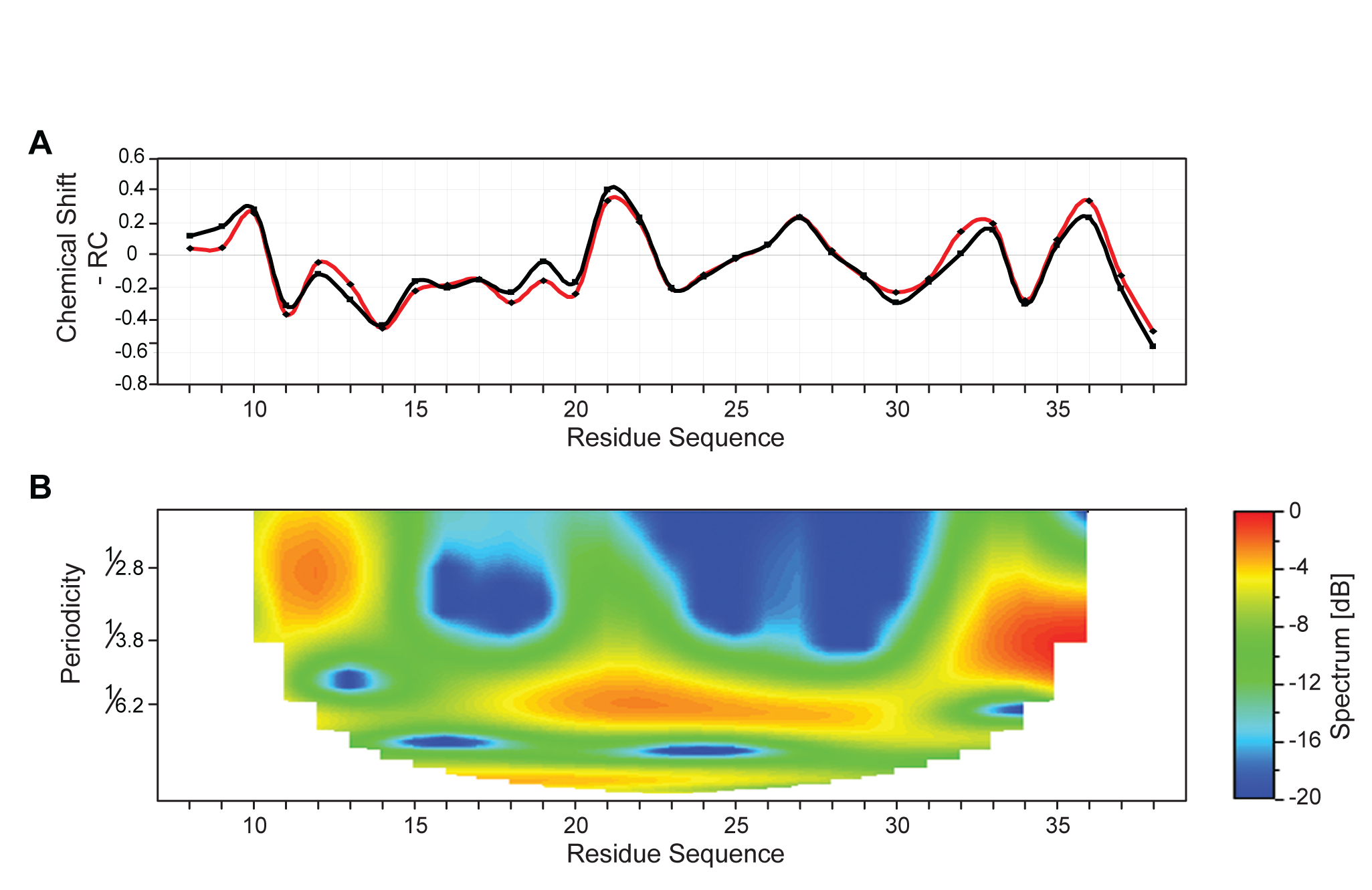

Supplement: Figure S4 — (A) Deviation of 1HN chemical shifts from random coil values in ETM (black trace). (B) Wavelet analysis of ETM amide chemical shifts shown in (A) yielded three types of periodicities as indicated by red/orange regions: the N-terminal, central and C-terminal regions showed periodicities (1/n) of 1/2.8, 1/6.2 and 1/3.8 residues, where number n indicates the number of residues required to complete a cycle in chemical shift variation. In (A), the red trace shows the of 100 mM AMT; the data for 10 mM HMA was similar (not shown). (8.21 MB TIF) [file ppat.1000511.s004.tif]

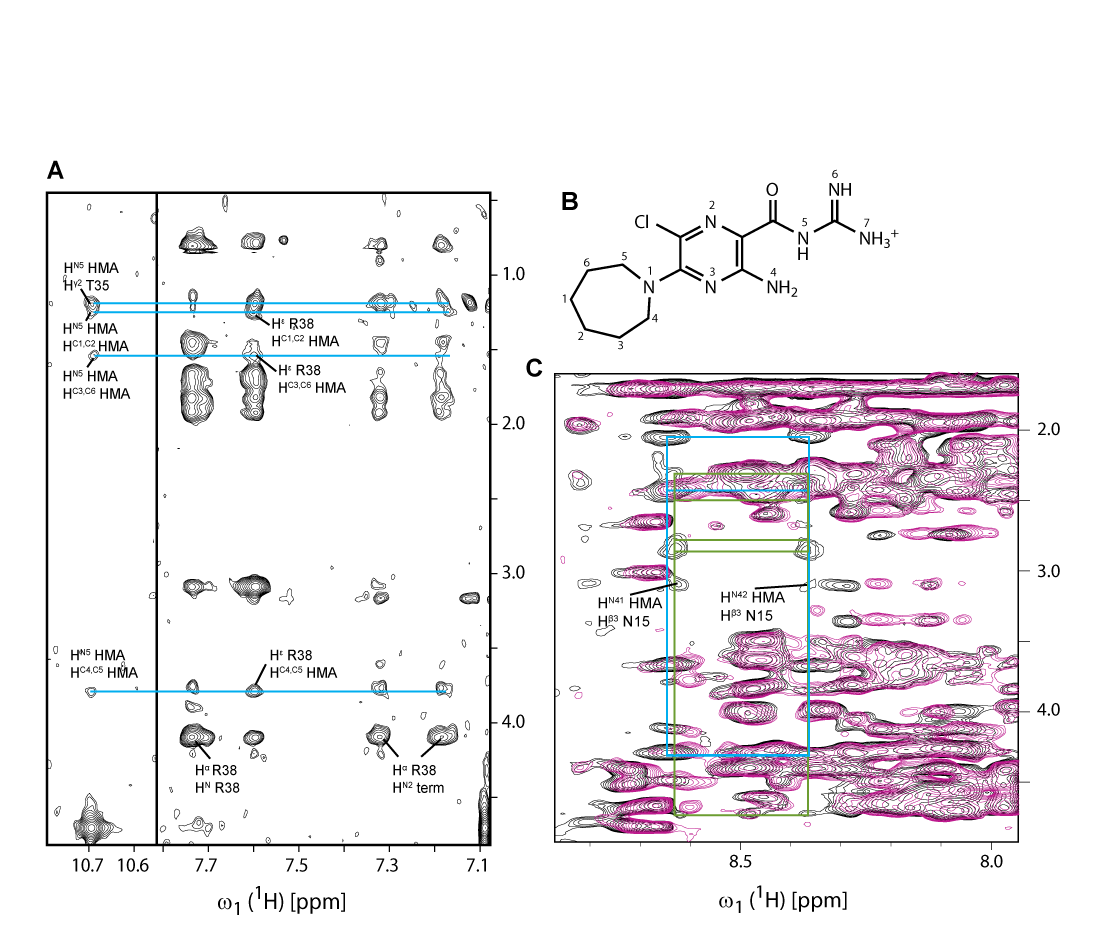

Supplement: Figure S5 — (A) 2D 1HN, 1Haromatic band-selected NOESY spectra of ETM in the presence of 10 mM HMA. The assignment of ETM and HMA resonances, as well as NOEs between the protein and HMA (blue lines) are indicated. (B) HMA molecule, with atoms numbered as in the Protein Data Bank (PDB) structure. (C) 2D 1HN, 1Haromatic band-selected NOESY and 2D water gate NOESY spectra of ETM in the absence (magenta) and presence (black) of 10 mM HMA. The assignment of two sets of connected spin systems from HMA bound at the N- and C-termini of ETM are indicated by green and blue lines, respectively. The assignment of selected NOEs is indicated. (3.14 MB TIF) [file ppat.1000511.s005.tif]

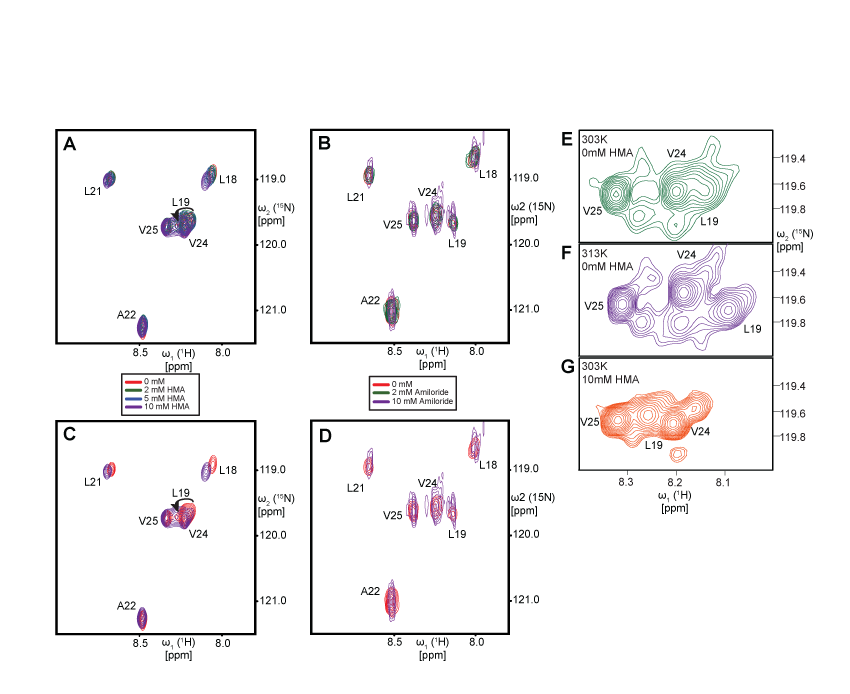

Supplement: Figure S6 — [1H,15N]-TROSY of ETM in DPC titrated at the final concentrations indicated (inserts) with (A) HMA at 30°C (B) amiloride at 37°C. (C) Same as in (A), but showing only two conditions: no drug and highest drug concentration tested. In (A) and (C), the shift for L19 is indicated by an arrow. (D) Same as (C), but for amiloride instead of HMA. No significant change in peak position is seen for amiloride at the highest concentration tested. (E) A fragment of the [1H,15N]-TROSY of ETM in DPC, comprising cross-peaks of Leu 19, Val 24 and Val 25, measured at 30°C; (F) the same at 37°C; (G) the same but in the presence of HMA, at 30°C. Results using AMT were similar to those with HMA (not shown). (1.80 MB TIF) [file ppat.1000511.s006.tif]

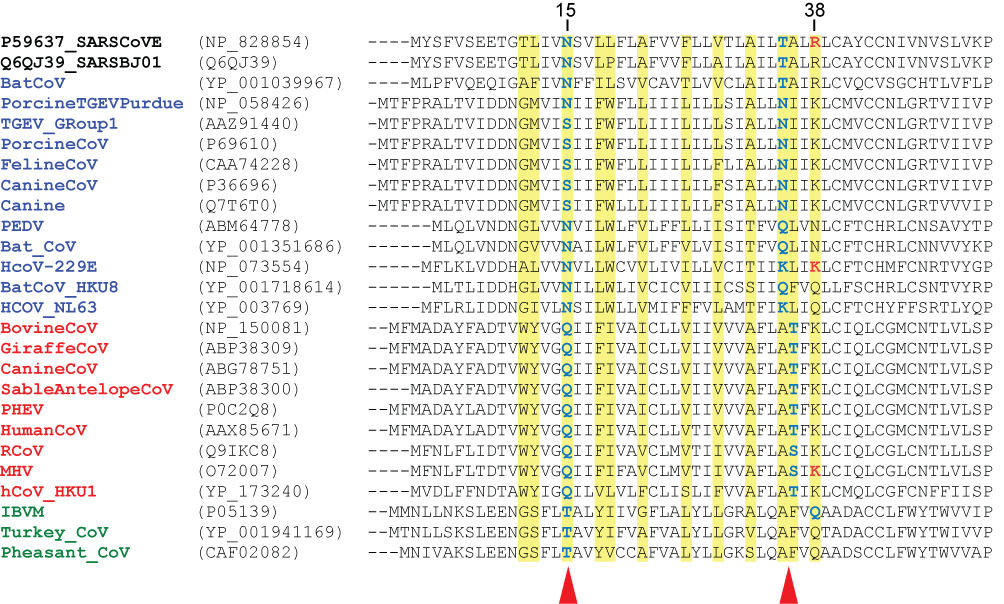

Supplement: Figure S7 — Clustal X sequence alignment of envelope proteins in coronaviruses, up to the totally conserved Pro residue (P54 in SARS-CoV E), corresponding to SARSCoV sequences (group 2b, black), group 1 sequences (blue), group 2 sequences (red) and group 3 sequences (green). The accession numbers are indicated next to the common name, on the left. The positions N15 and R38 in the SARS-CoV E sequence are indicated above, and the residues that are exposed to the lumen of the pore in ETM are shown with a yellow background. The locations of the two HMA binding sites in the ETM channel are indicated by a red arrow. (1.86 MB TIF) [file ppat.1000511.s007.tif]

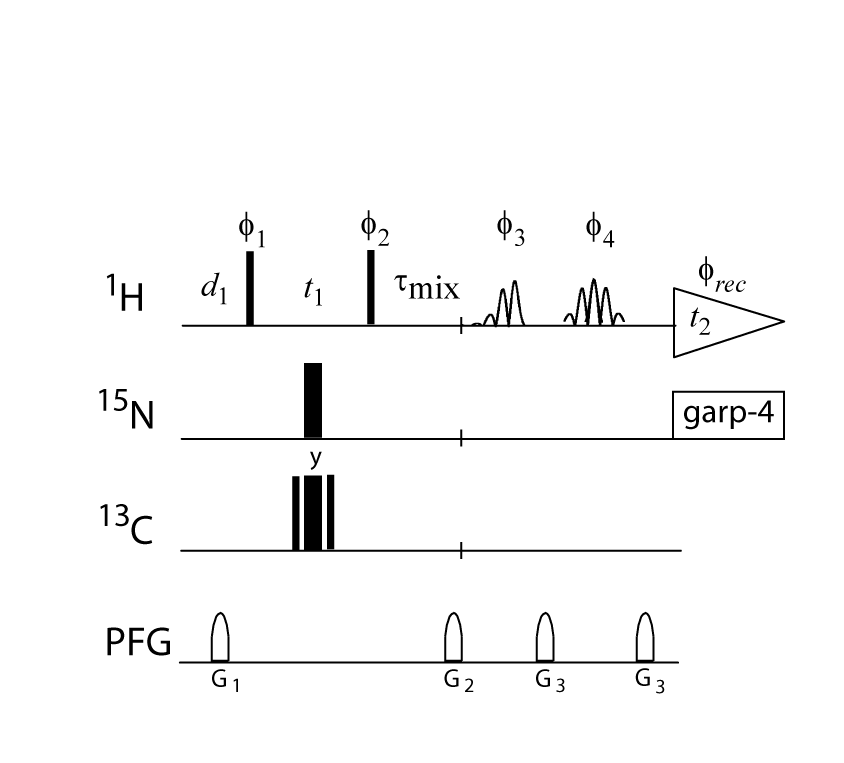

Supplement: Figure S8 — Scheme of 2D 1HN, 1Haromatic band-selected NOESY, an experiment suitable for detection of 1HN, 1Haromatic resonances in membrane proteins in the presence of strong aliphatic resonances of solubilizing detergents. Longitudinal relaxation acceleration scheme [72] prevents saturation of longitudinal magnetization of aliphatic spins and water building up during the mixing period τmix. This magnetization is used to accelerate relaxation of amide and aromatic protons to steady-state Boltzmann thermal equilibrium during the inter-scan delay d 1. The radiofrequency pulses on 1H, 15N, 13C are applied at 4.7, 118 and 40 ppm, respectively. Narrow and wide black bars indicate non-selective π/2 and π rf-pulses applied with the phase x unless indicated otherwise. Complex shapes on the line marked 1H indicate the 1HN, 1Haromatic band-selective 1.5 ms excitation E-Burp2 pulses with the phase ϕ3 and ϕ4 and γB1 = 2733 Hz and the 1.8 ms refocussing Re-Burp pulse [73] with the phase ϕ4 and γB1 = 3050 Hz. The center of the excitation of all 1HN, 1Haromatic bandselective pulses is placed at 8.5 ppm. The durations and strengths of the pulsed magnetic field gradients (PFG) applied along the z-axis are selected as G1: 500 µs, 80 G/cm; G2: 900 µs, 60 G/cm; G3: 900 µs, 70 G/cm. Two datasets, with and without 13C-composite inversion pulse decoupling pulse are acquired using the phases ϕ1 = x; ϕ2 = x; ϕ3 = x, y); ϕrec = (x, −x). The quadrature detection in t 1 dimension is achieved by the States-TPPI method [74] applied to ϕ1. Subtraction of the datasets results in a 2D NOESY spectrum containing NOEs stemming from 1H covalently bound to 13C spins and the other proximal 1H spins. (0.66 MB TIF) [file ppat.1000511.s008.tif]
