# Supplementary material for: Structure and Inhibition of the SARS Coronavirus Envelope Protein Ion Channel
Source: PLoS Pathog. 2009 Jul 10;5(7):e1000511. doi: 10.1371/journal.ppat.1000511 (PMC2702000; doi:10.1371/journal.ppat.1000511)
Supplement: Table S1 — Statistics of ETM structure reconstruction, alone or in the presence of amantadine and HMA. (0.06 MB DOC) [file ppat.1000511.s009.doc]

**Table S1**. Statistics of ETM structure reconstruction, alone or in the presence of amantadine and HMA.

|  | ETM | ETM amantadine | ETM  HMA |
| --- | --- | --- | --- |
| **NMR distance and dihedral constraints** |  |  |  |
| Distance constraints |  |  |  |
| Total NOE | 492 | 478 | 488 |
| Intra-residue | 82 | 122 | 126 |
| Inter-residue |  |  |  |
| Sequential (|*i* – *j*| = 1) | 342 | 310 | 316 |
| Medium-range (|*i* – *j*| < 4) | 66 | 46 | 46 |
| Long-range (|*i* – *j*| > 5) | 0 | 0 | 0 |
| Intermolecular/ monomer | 9 | - | 5 |
| Total dihedral angle restraints |  |  |  |
| φ | 31 | 31 | 31 |
| ψ | 31 | 31 | 31 |
|  |  |  |  |
| **Structure statistics** |  |  |  |
| Violations (mean and s.d.) |  |  |  |
| Distance constraints (Å) | 0.7 ± 0.1 | 0.6 ± 0.1 | 1.1 ± 0.1 |
| Dihedral angle constraints (º) | 0.0 ± 0.0 | 0.0013 ± 0.0040 | 0.0014 ± 0.0043 |
| Max. dihedral angle violation (º) | 0.0 | 0.0139 | 0.0149 |
| Max. distance constraint violation (Å) | 0.8 | 0.7 | 1.3 |
| Average pairwise r.m.s. deviation** (Å) |  |  |  |
| Heavy | 0.97 ± 0.29 | 0.67 ± 0.23 | 0.64 ± 0.24 |
| Backbone | 1.43 ± 0.26 | 1.13 ± 0.22 | 1.09 ± 0.20 |
| Ramachandran Analysis  Residues in favoured regions  Residues in additionally allowed regions  Residues in generously allowed regions  Residues in disallowed regions | 100.0 %  0.0 %  0.0 %  0.0 % | 99.1 %  0.9 %  0.0 %  0.0 % | 99.6 %  0.4 %  0.0 %  0.0 % |
