# Supplementary material for: Structure and Inhibition of the SARS Coronavirus Envelope Protein Ion Channel
Source: PLoS Pathog. 2009 Jul 10;5(7):e1000511. doi: 10.1371/journal.ppat.1000511 (PMC2702000; doi:10.1371/journal.ppat.1000511)
Supplement: Table S2 — Inter-helical NOEs for ETM derived from difference 2D homonuclear 1HN, 1Haromatic band-selected NOESY. (0.04 MB DOC) [file ppat.1000511.s010.doc]

**Table S2**. Inter-helical NOEs for ETM derived from difference 2D homonuclear ^1^H^N^, ^1^H^aromatic^ band-selected NOESY.

| From | | To | Interaction |
| --- | --- | --- | --- |
| L18 | ^1^H_3_^δ1^ | ^1^H^δε^ phenyl ring (F23) | Strong |
|  | ^1^H_3_^δ2^ | ^1^H^δε^ phenyl ring (F23) | Strong |
| L21 | ^1^H_3_^δ1^ | ^1^H^δε^ phenyl ring (F23) | Strong |
|  | ^1^H_3_^δ2^ | ^1^H^δε^ phenyl ring (F23) | Strong |
|  | ^1^H_3_^δ2^ | ^1^H^δε^ phenyl ring (F26) | Weak |
