# Supplementary material for: Structure and Inhibition of the SARS Coronavirus Envelope Protein Ion Channel
Source: PLoS Pathog. 2009 Jul 10;5(7):e1000511. doi: 10.1371/journal.ppat.1000511 (PMC2702000; doi:10.1371/journal.ppat.1000511)
Supplement: Table S3 — Assignment of two forms of HMA bound to the N- and C-termini of ETM. (0.04 MB DOC) [file ppat.1000511.s011.doc]

**Table S3.** Assignment of two forms of HMA bound to the N- and C-termini of ETM.

**Form A (bound at C-terminus)**

H2(C1) and H2(C2) 1.54 ppm
H2(C3) and H2(C6) 1.80 ppm
H2(C4) and H2(C5) 3.80 ppm
H1(N4) - 8.65 ppm
H2(N4) - 8.36 ppm
H(N5) - 10.69 ppm

**Form B (bound at N-terminus)**

H2(C1) and H2(C2) 1.81 and 1.99 ppm
H2(C3) and H2(C6) 2.27 and 2.33 ppm
H2(C4) and H2(C5) 4.21 ppm
H1(N4) - 8.63 ppm
H2(N4) - 8.37 ppm

H(N5) – not visible as a separate signal
