# Supplementary material for: Structure and Inhibition of the SARS Coronavirus Envelope Protein Ion Channel
Source: PLoS Pathog. 2009 Jul 10;5(7):e1000511. doi: 10.1371/journal.ppat.1000511 (PMC2702000; doi:10.1371/journal.ppat.1000511)
Supplement: Protocol S1 — Supplementary methods. (0.02 MB PDF) [file ppat.1000511.s012.pdf]

METHODS

Orientation of helices with respect to the lipid phase probed by paramagnetic relaxation.

Longitudinal relaxation of $^1H^N$ spins enhanced by the proximity to a paramagnetic probe can be used to map the orientation of helices respect to the lipid phase[1]. Due to the compact arrangement of the six ^{15}N-labeled amino acids near the center of the transmembrane helices, the paramagnetic relaxation enhancement (PRE) should reflect the distance of the corresponding $^1H^N$ to the surface of the pentameric assembly, provided the employed paramagnetic probe is primarily localized at the center of a micelle. We selected a 16-DSA, which typically induces PRE near the center of DPC micelles carrying a membrane protein[2].

Following the theoretical guidelines outlined by Zangger and co-workers[1], we derive below an analytical expression for the dependence of PRE on the shortest distance of $^1H^N$ to the lipid-exposed outer surface of the ETM channel. For paramagnetic probes where the unpaired electron has a short longitudinal relaxation time, the influence of the unpaired electron on nuclear relaxation (both longitudinal and transverse) can be described by the “outer sphere” relaxation approach [3]. In order to obtain an expression for PRE as a function of geometric parameters, we performed a volume integration shown in Equation (1). In this equation, the probability of finding a paramagnetic center in a particular volume element of the system formed by ETM, detergent and 16-DSA, is multiplied by the inverse of the 6th power of the proton-electron distance, r, and integrated[1].

$$PRE = \iiint_V \frac{e^{-\frac{r}{aR}}}{r(x,y,z)^6} dxdydz \tag{1}$$

To ETM in DPC, we added the lipid analogue 16-DSA, bearing a doxyl paramagnetic radical (spin electron) at position 16 of the alkyl chain. This resulted in a measurable PRE effect that was linearly proportional to the concentration of 16-DSA in the range 1 mM to 5 mM.

In this calculation, the ETM system can be described as a pentameric α-helical bundle (Fig. S3, A), represented by a cylinder with radius $\boldsymbol{R_b}$, aligned with the z-direction of the laboratory coordinate frame. This cylinder is embedded in a spherical micelle of radius ***R***, and integration should be limited to the volume of the micelle accessible to the probe, ***V.*** This volume comprises the core and inner layers of the micelle, i.e., a fraction ***a*** of the total micelle volume. The nuclear spin is localized at the immersion depth ***d*** (Fig. S3, B) from the surface of the bundle accessible to the paramagnetic probe. For the sake of simplicity, Eq. (1) can be approximated as PRE = $\mathbf{a}/d^3$, where **a** is a collection of various fundamental constants. $\boldsymbol{R_b}$ is defined by a rolling-sphere approach, with sphere radius of 3 Å, corresponding to the size of the doxyl radical (Fig. S3, D).

Alternatively, PRE can be calculated using a distance from the amide proton to the axis of the pentamer (Fig. S3, C). The position of this axis can be defined with greater precision than the immersion depth from the surface of the pentamer. In this case the equation can be simplified to PRE = $\mathbf{a}/(D/2\text{-}l)^3$, where **a** is a collection of various fundamental constants, *D* is the diameter of the cylinder representing the pentamer and *l* is the distance from the C_5 symmetry axis to the amide proton.

1. Respondek M, Madl T, Gobl C, Golser R, Zangger K (2007) Mapping the orientation of helices in micelle-bound peptides by paramagnetic relaxation waves. J Am Chem Soc 129: 5228-5234.
2. Hilty C, Wider G, Fernandez C, Wuthrich K (2004) Membrane protein-lipid interactions in mixed micelles studied by NMR spectroscopy with the use of paramagnetic reagents. Chembiochem 5: 467-473.
3. Bertini I, Fragai M, Luchinat C, Parigi G (2001) Solvent H-1 NMRD study of hexaaquochromium(III): Inferences on hydration and electron relaxation. Inorganic Chemistry 40: 4030-4035.
